# Hartmann’s procedure reversal rate and short-term outcomes: a single centre retrospective cohort study

**DOI:** 10.1186/s12893-026-03738-x

**Published:** 2026-04-30

**Authors:** Christopher C L Liao, Balakrisanan Saravanan, Fatima Ismail, Matthew Farmer, Vamsi R Velchuru

**Affiliations:** 1 Department of Colorectal & General Surgery, James Paget University Hospitals NHS Foundation Trust, Lowestoft Road, Gorleston, Great Yarmouth, Norfolk, NR31 6LA United Kingdom; 2 Department of Emergency Medicine, James Paget University Hospitals NHS Foundation Trust, Lowestoft Road, Gorleston, Great Yarmouth, Norfolk, NR31 6LA United Kingdom

**Keywords:** Hartmann’s procedure, Hartmann’s reversal, Open surgery, Laparoscopy, Morbidity, Mortality

## Abstract

**Background:**

Hartmann’s procedure is when all the sigmoid and/or rectum is removed with an end stoma in the left iliac fossa and the rectal stump closed off in the pelvis. This is often performed when bowel anastomosis is not feasible, too risky or deemed unsuitable for patients, either as planned operation or in an emergency, with reversal rate being less than 50%. The aim of our study was to establish the number of Hartmann’s procedure performed, its reversal rate in a single UK based NHS hospital, comparison between surgical approaches (laparoscopic vs. open) and evaluating short term clinical outcomes.

**Methods:**

A retrospective cohort study was conducted over a period of 13 years and 4 months (1^st^ Jan 2011–31^st^ May 2024). Exposures were (1) reversal status (reversed vs. non-reversed) and (2) surgical approach to reversal (laparoscopic vs. open). Patient demographics including smoking status, comorbidities, ASA, Rockwood frailty score, WHO performance status, and surgical indication were extracted. Outcomes included 30-day Clavien-Dindo morbidity, anastomotic leak, return to theatre, length of stay, readmission rate, and 30 and 90-day mortality.

**Results:**

A total of 172 patients underwent Hartmann’s procedure during the study period, of whom 90 (52.3%) were reversed. The median follow-up was 67.25 months (IQR +/- 87). Forty-seven patients died during follow-up, 38 (81%) of whom were in the non-reversed group. Patients undergoing reversal were significantly younger, with fewer comorbidities, better ASA, frailty, and performance scores. Of the 90 reversals, 25 were completed laparoscopically, 35 were converted from laparoscopic to open, and 30 were performed as planned open reversals. Laparoscopic reversal was associated with a significantly shorter median length of stay (+/- 4 days); however, there was no difference in 30-day Clavien-Dindo morbidity between groups. There were two anastomotic leaks (2.2%) and two failed reversals in the open group, and one 30-day mortality in the laparoscopic group.

**Conclusions:**

Our single-centre UK experience shows higher rates of Hartmann’s reversal in younger, much fitter patients, shorter hospital stay in the laparoscopic reversal group, with acceptable short-term outcomes in both groups.

## Introduction

 Since described by Henri Hartmann in 1921 at a French Surgical Congress, Hartmann’s procedure (HP) involves resection of the rectosigmoid colon with formation of an end-colostomy and closure of the rectal stump [1]. Due to its non-restorative nature HP is often performed as an emergency due to patient’s presentation and intra-abdominal contamination when bowel anastomosis is deemed too risky or not in the patient’s best interest [2, 3]. Planned HP could often be performed for non-obstructing malignancy, in frail patients as a palliative procedure when permanent stoma is the only option, including those patients with poor sphincter function [4] Although primary anastomosis is increasingly favoured, particularly for complicated diverticulitis [5–8], where evidence suggests improved short- and long-term outcomes, better stoma reversal rate and being more cost-effective [9], HP continues to play an important role in emergent patients and those with high operative risk.

However, reversal of Hartmann’s procedure (HR) remains complex as it is still considered a technically challenging operation compared to an elective left colectomy [10] and many published case series show a reversal rate of < 50% in long term follow-up [6, 11]. This is due to either patients’ refusal, advanced age or significant co-morbidities with poor performance status. Factors influencing likelihood of reversal include younger age, benign pathology, lower ASA grade and comorbidity index, and preserved rectal stump length [11–15]. Minimally invasive techniques have been increasingly adopted for HR, with laparoscopic reversal associated with shorter hospital stay, reduced postoperative morbidity, and quicker recovery compared to open approaches [16–18].

While previous studies of HR come from high-volume/ tertiary centres, there is limited published data on outcomes from smaller hospitals where operative case-mix, resources, and operative volumes may differ significantly. Our study addresses this gap by presenting a carefully conducted retrospective review of all HPs performed at our institution over a 13-year period, analysing reversal rates, patient characteristics, and perioperative outcomes. We specifically compare laparoscopic versus open reversal, survival length following reversal, hospital stay, and short-term morbidity and mortality. By reporting data from a 450-beded UK based district general hospital (DGH), we aim to provide benchmark evidence to guide clinical decision-making in similar healthcare setup.

## Materials and methods

### Study design

This was a retrospective, observational study following an audit to collect the data on all consecutive patients who underwent HP in a single UK based district general hospital, over a period of 13 years and 4 months starting from 1st January 2011 till 31st May 2024. Retrospective data was collected from prospectively maintained patient notes such as electronic patient’s health records. All patients who had HP whether eligible or not for subsequent reversal was considered. HP was considered as elective, planned anterior resection or emergency sigmoidectomy where part of rectum or all the sigmoid is removed with an end colostomy fashioned in the left iliac fossa and closure of the rectal stump. The decision for HP was made by the attending surgeon as a planned procedure (elective) or in an acute setting, where such procedures were done laparoscopically or an open approach depending on the surgeon’s skills, intra-operative findings and the feasibility of removing the diseased segment. The decision to mobilise the splenic flexure was made by the attending surgeon based on the length of the exteriorised colon and the distance of the pathology from the closed off rectal stump. The decision of high-tie ligation of the inferior mesenteric artery (IMA) was also made by the attending surgeon dependent on the pathology encountered. High tie of the IMA was performed in cancer related pathology but often preserved if it was due to a benign condition. However, all reversal was performed as a planned operation only by colorectal trained surgeons either laparoscopically or open, based on their primary surgery and intraoperative findings, presence of adhesions on scope insertion and access to the rectal stump. All patients undergoing reversal were thoroughly assessed in clinic prior to planned surgery with endoscopic evaluation of their bowel, radiological scan wherever necessary along with pre-operative bloods and anaesthetic assessment. Post-operatively patients were cared for in the ward in line with institutional enhanced recovery after surgery (ERAS) protocol and only discharged home when patients were independently mobilising, managing an oral diet and following a satisfactory bowel movement.

### Data collection

**Fig. 1:**
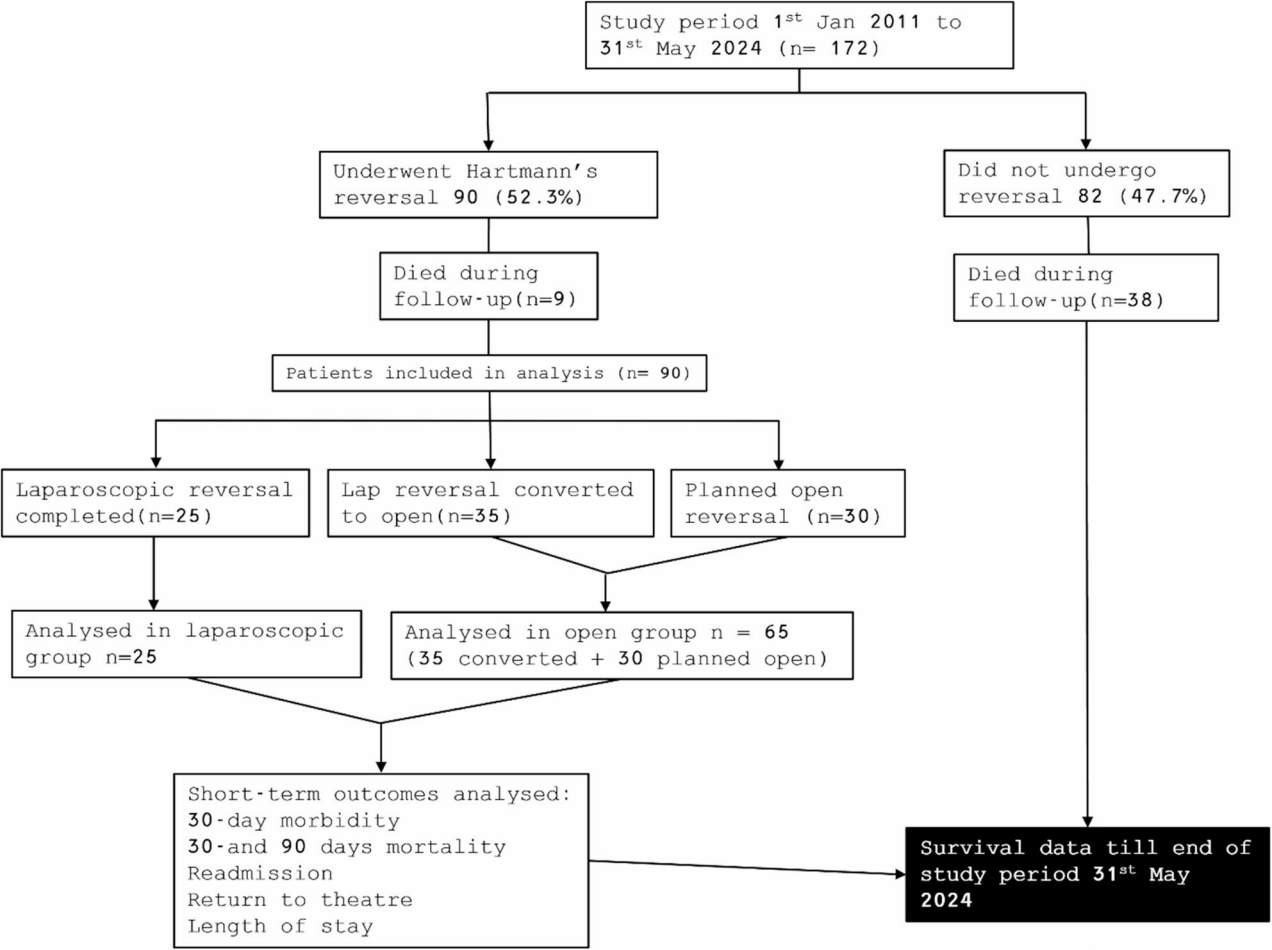
STROBE flow chart. A total of 172 patients underwent a Hartmann’ procedure, out of which 90 underwent a Hartmann’s reversal and 82 did not. Total of 47 patients died during the study period, out of which 9 belonged to the reversal group and 38 in the group that was not reversed. All 90 patients who underwent reversal were analysed based on the operative approach whether laparoscopic, conversion to open or open reversal and their short-term outcomes along with survival data until the end of study period on 31^st^ May 2024

Data was collected for a total of 172 patients who underwent Hartmann’s procedure during this period, out of which, at the time of review, 90 patients had undergone reversal; 47 patients had died out of which 38 patients belonged to the group that was not reversed as shown in the STROBE flowchart (Fig. 1). Data was collected on demography including Body Mass Index (BMI), co-morbidities, ASA (American society of Anaesthesiologists) score, smoking status, WHO performance status, Rockwood frailty score, surgical approach and urgency to Hartmann’s procedure, indication for surgery, interval days before reversal. Data was also collected on those who underwent Hartmann’s reversal based on demography, surgical approach for reversal, operative time, length of stay, early post-operative complications such as 30-days Clavien-Dindo classified morbidity, re-admission within 30 days, leak rates, return to theatre, and 30- & 90-days mortality. All the data was collected from prospectively maintained electronic and paper chart medical records including operative details from theatre database.

### Ethical approval

Ethical approval for this study was waived by the Institutional Research Board of the James Paget University Hospitals NHS Foundation Trust, in view of its retrospective design. The study was conducted as part of a departmental audit, utilising data retrospectively extracted from routinely maintained prospective electronic medical records. No identifiable patient information was accessed, and therefore individual informed consent was not required. All data were handled in full compliance with the General Data Protection Regulation (GDPR) and institutional policies on research governance and data confidentiality. The study adhered to the ethical principles outlined in the Declaration of Helsinki (1964) and its subsequent amendments.

### Statistical analyses

A descriptive statistical analysis was done to compare the Hartmann’s reversal group with the group that was not reversed. Shapiro-Wilk test or Kolmogorov-Smirnov test was performed to determine whether the data was normally distributed or not. Median with interquartile range was used for non-parametric data. Mann-Whitney U test or Kruskal-Wallis test was used for continuous data, Chi-square test or the Fisher exact test was used for categorical data. A binomial logistic regression model was used to determine the predictors of Hartmann’s reversal. A Kaplan-Meier Curve was also plotted for all patients who underwent reversal vs. those who did not undergo reversal for the number of days patients were alive since the Hartmann’s procedure to the end of the study period of 31^st^ May 2024.

Finally, descriptive statistical analysis was done to compare the outcome results between Laparoscopic and Open approach of all those who underwent Hartmann’s reversal using an as-treated protocol analysis, with those patients who were converted to open from laparoscopy, in the open group. All data was analysed using SPSS Ver 29 (IBM SPSS Inc., Chicago, Illinois, USA). A *p*-value of < 0.05 was considered statistically significant in all our analyses.

## Results

All together during the study period from 1st Jan 2011 till 31^st^ May 2024, there was 172 Hartmann’s procedure performed at a single centre UK District general hospital, out of which 90 (52.3%) patients were reversed and 82 (47.7%) were not reversed. The baseline demography of all the patients who had HP within the study period, along with those reversed vs. not reversed is shown in Tables 1 and 2. Those who underwent HR were significantly younger and fitter with lower Charlson’s co-morbidity index, lower ASA status, better WHO performance status and lower Rockwood frailty score when compared to those who did not undergo reversal. There was no difference in gender ratio, smoking status and body mass index (BMI). There were more emergency cases done for benign conditions through an open approach, compared to elective cases performed for malignancy through laparoscopic approach in the reversed group, whereas equal number of benign and malignant cases performed equally as emergency and elective procedures in the non-reversed group.

**Table 1:** Baseline demographics and fitness characteristics of all patients with comparison between ‘reversed’ and ‘not reversed’ groups

	All patients (*n* = 172)	Reversed (*n* = 90)	Not reversed (*n* = 82)	*p* value
Age (median +/- IQR)	64 years (IQR 22.25)	56.5 years (IQR 17.7)	75 years (IQR 14.0)	< 0.0001
Gender (M: F)	94/ 78	51/ 39	43/ 39	0.687
Smoking status (current/ ex/ non-smoker)	48/ 64/ 60	25/37/28	23/27/32	0.462
Charlson’s co-morbidity index (CCI)	3.0 (IQR 1 to 5)	2.0 (IQR 1 to 3)	5.0 (IQR 3 to 6)	< 0.0001
Body Mass Index (BMI)	26.5 (IQR 7.0)	26.85 (IQR 6.19)	26.2 (IQR 7.65)	0.1797
ASA 1	7	6	1	< 0.0007
2	79	55	24	
3	83	29	54	
4	3	0	3	
Frailty score (Median IQR)	2.0 (IQR 1.0)	2.0 (IQR 0.0)	3.0 (IQR 1.0)	< 0.0001
WHO performance status	1 (IQR 1.0)	0 (IQR 0)	0 (IQR 1)	0.004
Elective vs. Emergency	44/ 82	10/ 51	34/ 31	< 0.0001
Diagnosis (Benign vs. Malignant)	118/ 54	76/ 14	42/ 40	< 0.001
Hartmann’s approach (Lap/ Lap to open & Open)	37/ 13/ 122	11/ 4/ 75	26/ 9/ 47	< 0.001

**Table 2:** Index Hartmann’s procedure characteristics

	All patients (*n* = 172)	Reversed (*n* = 90)	Not reversed (*n* = 82)	*p* value
Elective vs. Emergency	44/ 82	10/ 51	34/ 31	< 0.0001
Diagnosis (Benign vs. Malignant)	118/ 54	76/ 14	42/ 40	< 0.001
Hartmann’s approach (Lap/ Lap to open & Open)	37/ 13/ 122	11/ 4/ 75	26/ 9/ 47	< 0.001

The data showed better odds of being reversed if HP was performed as an emergency for benign cases through an open approach, compared to fully laparoscopic or laparoscopy converted to open cases (Table 3). A multivariable logistic regression analysis was performed to adjust for confounders and identify independent predictors of reversal. The model showed significantly reduced odds of reversal with increasing age (OR 0.91), higher Charlson’s co-morbidity index (OR 0.54), poorer WHO performance status (OR 0.52), higher frailty score (OR 0.26) & ASA grade (OR 0.24) and diagnosis of Malignancy, while emergency surgery for benign disease and open approach significantly increased the odds of reversal (OR 5.9 and OR 3.72 respectively). These findings suggest that reversal likelihood is largely driven by patient fitness and surgical context rather than baseline demographics alone.

**Table 3:** Predictors for reversal of Hartmann’s procedure

Parameters	Odds ratio (95% CI)	*P* value
Age	0.91 (0.87–0.93)	*P* < 0.001
Charlson’s co-morbidity index (CCI)	0.54 (0.44–0.64)	*P* < 0.001
WHO performance status	0.52 (0.33–0.81)	*P* < 0.001
Frailty score	0.26 (0.15–0.42)	*P* < 0.001
ASA grade	0.24 (0.12–0.42)	*P* < 0.001
Emergency vs. Elective	5.9 (2.79–12.43)	*P* < 0.001
Cancer	0.19 (0.095–0.396)	*P* < 0.001
Diverticulitis perforation	3.58 (1.87–6.84)	0.0046
Hartmann’s approach (Open/ Lap/ Lap to Open)	3.72 (1.83–7.54)	*P* < 0.001

**Fig. 2:**
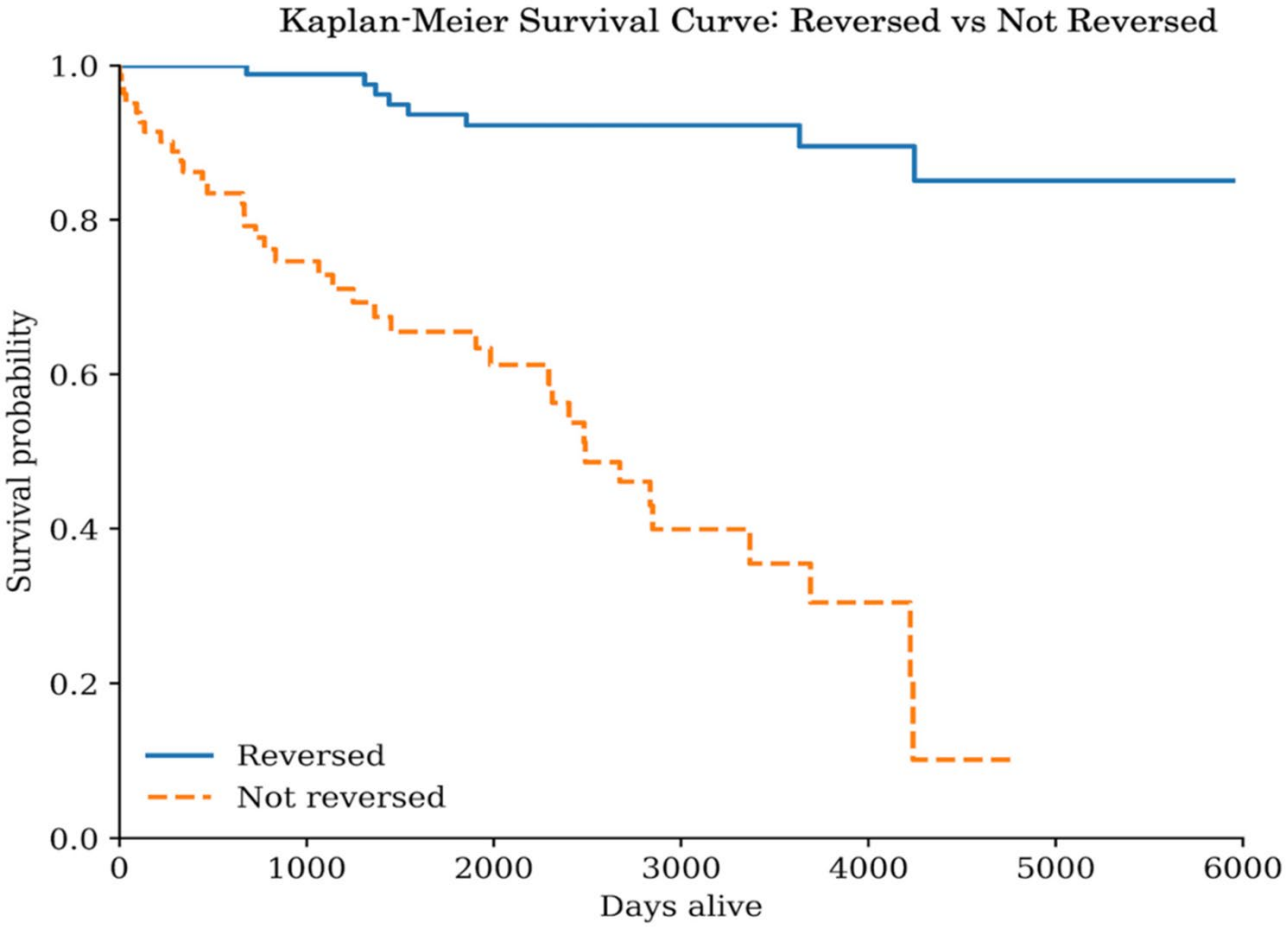
A Kaplan-Meier Curve for Hartmann’s reversal vs. those who did not undergo reversal plotting the number of days alive since the Hartmann’s procedure to the end of the study period on 31^st^ May 2024

The number of days patients were alive following a HP till the end of the study period on 31^st^ May 2024, plotted on a Kaplan-Meier chart between those who were reversed vs. not-reversed is shown in Fig. 2. In the reversed Group, median number of days alive: 2860 days (approx. 7.83 years) IQR +/- 2122 days and in the Group not reversed, the median number of days alive: 1101 days (approx. 3.02 years) IQR +/- 2014 days. The difference between the groups favours the reversal group showing a significantly longer survival duration (*p* < 0.00001).

**Fig. 3:**
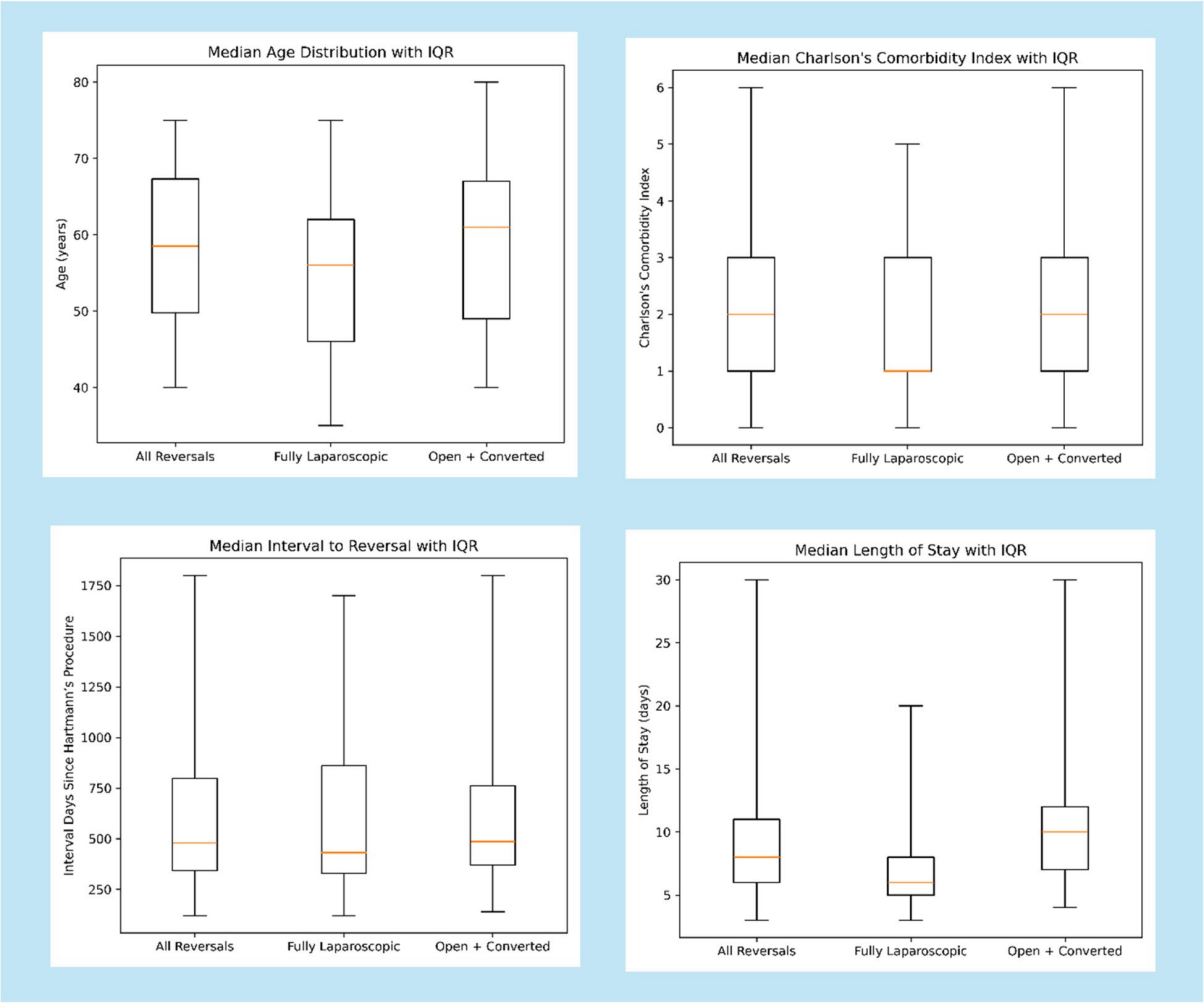
Graphical representation of Age, Charlson’s comorbidity index, Interval days from HP to reversal and median Length of Stay shown for all Hartmann's reversals, fully laparoscopic group and the open group with conversions in box and whiskers plot

All those cases that were reversed were analysed on as-treated basis by comparing fully laparoscopic group with the open group that included the converted cases for analysis. Out of 55 attempted laparoscopic reversals, 35 (63.6%) required conversion to open surgery, giving a conversion rate of 39%. The overall results are shown in Table 4 and the graphical representation of Age, Charlson's comorbidity index, interval to reversal and median length of stay is shown in Fig. 3. There was no difference in age, gender, co-morbidities, ASA grade, Frailty score, WHO performance status, BMI, benign or malignant cases, interval days since HP and operative time between the laparoscopic vs. open HR group. The only significant difference was reduced length of stay by median of 4 days favouring laparoscopic reversal. Early post-operative morbidity within 30-days following HR based on the Clavien-Dindo classification was 38.5%, with majority seemed to have occurred in the open reversal group as shown in Table 5, but this difference did not reach statistical significance. The list of immediate 30-day complications occurring in the reversal group along with failure to reverse, return to theatre, 30-day readmission rate with mortality is listed in Table 6. There were two anastomotic leaks and two failures to reverse both in the open group, and one death in this post-operative period in the laparoscopic group. The incidence of early post-op morbidity based on Clavien-Dindo classification does not show any significant difference between the two groups, even though the wound infection rate appears to be lower in the fully laparoscopic group, but this finding did not reach significance, due to small numbers (Figs. 2 and 3).

**Table 4:** Patient characteristics, perioperative data and length of stay (LOS) for all Hartmann’s reversal (*n* = 90)

	Total patients reversed (*n* = 90)	Fully Laparoscopic (*n* = 25)	Open + Lap converted to open (*n* = 30 + 35)	*p* value
Age	58.5 years (IQR 49.8–67.3)	56 years (IQR 46–62)	61 years (IQR 49–67)	0.543
Gender (M: F)	51: 39 (1.31: 1)	18: 7 (2.57: 1)	33: 32 (1.03: 1)	0.611
Charlson’s Co-morbidity Index (CCI)	2 (IQR 1–3)	1 (IQR 1–3)	2 (IQR 1–3)	0.324
ASA grade	2 (IQR 2–3)	2 (IQR 2–2)	2 (IQR 2–3)	0.148
Frailty score	2 (IQR 2–2)	2 (IQR 2–2)	2 (IQR 2–3)	0.889
WHO performance status	0 (IQR 0–0)	0 (IQR 0–0)	0 (IQR 0–1)	0.080
BMI	26.85 (IQR 6.2)	26.8	24.5	0.076
Diagnosis – Benign	76	22	54	0.512
- Malignant	14	3	11	0.801
Interval days since Hartmann’s procedure	480 days (IQR 342–798)	432 days (IQR 329–860)	486 days (IQR 371–762)	0.836
Operative time	244.5 min (IQR +/- 115)	193 min (IQR +/- 61)	205 min (IQR +/- 62)	0.221
Length of Stay (LOS)	Median 8 days (IQR 6–11)	Median 6 days (IQR 5–8)	Median 10 days (IQR 7–12)	0.0027

**Table 5:** Early post-op (Clavien-Dindo grade complications) within the first 30-days following HR in 90 patients

Clavien-Dindo grade of complications	Reversed group (*n* = 90)		*p* value
Surgical approach	Lap reversal (*n* = 25)	Open (Converted + Open) *n* = 65	
Grade 1	1	6	0.668
Grade 2	4	18	0.288
Grade 3a	0	2	1.00
Grade 3b	1	0	0.278
Grade 4	0	4	0.573
Grade 5	0	0	1.00
Total cases	6 (6.6%)	30 (33.3%)	0.063

**Table 6:** Early post-operative Clavien-Dindo morbidity within 30 days in the Reversed group with failure to reverse, return to theatre, 30-day readmission and 90-days mortality

	Total patients reversed (*n* = 90)	Fully Laparoscopic (*n* = 25)	Open + Lap converted to open (*n* = 30 + 35)	*p* value
Clavien-Dindo morbidity within 30 days	Median 0.0 (IQR +/- 2.0)	Median (0.0 +/- 0.0)	Median (1.0 +/- 1.0)	0.064
Wound infection	20	2	18	0.0837
LRTI (chest infection)	10	2	8	0.083
Ileus	5	2	3	0.9
Atrial fibrillation	2	0	2	0.92
Anastomotic leak	2	0	2	0.92
Transfusion	2	0	2	0.91
Bleeding	1	0	1	1.0
Small bowel injury	1	0	1	0.61
Failure to reverse	2	0	2	0.92
Return to theatre	6	1	5	0.875
30-days readmission	8	1	7	0.55
90-days mortality	1	1	0	0.61

## Discussion

Our single centre experience shows a higher reversal rate of 52.3% consistent with the upper range of published series from 19% to 61% [6, 11, 14, 15, 19–21]. This is due to more benign cases operated as emergency through an open approach being reversed, compared to planned operation for malignancy, performed laparoscopically with the decision not to reverse, due to either poor sphincter function, advanced malignancy, or patients awaiting further chemotherapy [6, 15, 19]. Also, the patients who underwent reversal were significantly younger, with lower comorbidity index, ASA score and performance status. This would conform with previously published studies [11, 14] where patient aged < 69–70 were most likely to undergo reversal, as younger, fitter patients would more likely to be selected for HR, compared to older, frail patients with higher co-morbidities, who would chose not to undergo reversal, or may have higher risk of morbidity & mortality. Our study reflects the same as the median age of those reversed was significantly lower compared to those not reversed, supported by the Kaplan-Meier chart showing those who were reversed had a significantly longer median period of follow-up, and lower mortality than those not reversed. However, this apparent survival advantage also reflects selection bias, as patients undergoing reversal were younger, fitter, with better ASA scores rather than a causal effect of reversal itself.

A multi-variable analysis of all patient characteristics, surgical indication and approach, shows reducing odds of Hartmann’s reversal with advancing age, higher comorbidity index, poor WHO performance status, higher frailty and ASA score, but higher odds of being reversed for benign conditions performed through an open approach. The predictors of reversal identified in the regression analysis are likely modelling the decision-making process rather than identifying independent biological predictors. These findings are in keeping with previously published studies that HP performed as an emergency due to a benign conditions, is more likely to undergo reversal due to it being performed for relatively younger patients (compared to malignancy cases) [6, 15, 22] Many of these patients’ characteristics when incorporated into scoring systems can predict non-reversal with higher scores, as lower scores would often get reversed with better outcomes [23, 24].

Our analysis between laparoscopic vs. open, therefore showed no difference in terms of age, gender, comorbidity index, ASA grade, performance status and frailty score between the two groups. Our conversion rate of 39% was also high compared to many studies [25, 26] but within the reported range of 16% to 50% [16, 18] This is very likely due to the high proportion of index HP being performed by non-laparoscopic trained general surgeons on an emergent basis through an open approach, which likely contributed to more technically challenging laparoscopic reversal and a higher conversion rate. Although previous studies suggest that laparoscopic expertise confers an advantage [17] the success of laparoscopic reversal is multifactorial. Surgical decision-making for both the index HP and reversal procedures was not standardised and largely based on the attending surgeon’s expertise, intraoperative findings, and patient condition. While all reversals were performed by colorectal-trained surgeons, surgeon experience and operative preference were not standardised. Therefore, individual variation in laparoscopic expertise and institutional evolution of practice over time may have contributed to outcome differences. Also, robotic surgery was not available at our institution during the study period, therefore, the potential impact of robotics on HR could not be assessed and lies outside the scope of this study. Furthermore, case selection for reversal itself may limit causal interpretation of outcomes despite statistical comparisons. The use of an as-treated analytical approach may have introduced dilution bias and may attenuate the observed differences between laparoscopic and open reversal, particularly with respect to length of stay. However, this approach was chosen to better reflect real-world postoperative course experienced by patients, which is clinically most relevant when evaluating perioperative outcome, although this likely results in a conservative estimate of the true benefit of laparoscopic reversal.

Many studies advocate timing of reversal matters putting more significance to early reversal [11, 12, 27, 28]. Our study did not explore the benefits of early vs. later reversal as most of our patients in the National Health Service, UK (NHS) waited approximately the same median period of 16 months due to the nature of the NHS wait list [13]. The observed shorter length of stay in the laparoscopic group may reflect patient selection rather than a definitive procedural benefit, despite similar findings reported in previous studies [18, 20, 25, 29].

Comparing short-term outcomes, the wound infection rate was distinctly lower in the laparoscopic group, but this did not reach statistical significance due to small numbers. Previous meta-analysis has shown overall reduced morbidity associated with laparoscopic HR [18], such as reduced wound infection rates, incidence of paralytic ileus and reduced length of stay (LOS). Our study also demonstrated a shorter median LOS in patients who underwent laparoscopic reversal (6 days vs. 10 days, *p* = 0.0027), but this observed shorter LOS should not be interpreted as causal as the choice of surgical approach was not randomised and therefore, the reduced LOS observed in the laparoscopic group may reflect favourable patient selection rather than an intrinsic benefit of laparoscopy [29] and should be interpreted cautiously. Several outcomes were infrequent, limiting statistical power for between-group comparisons. The absence of statistical significance for outcomes such as wound infection should not be interpreted as evidence of equivalence. The observed difference may still be clinically relevant, but the small sample size and low event counts result in wide confidence intervals and an appreciable risk of type II error. For rare complications with only one or two events, the data are best interpreted descriptively rather than inferentially.

Our short-term results showed a lower rate of anastomotic leaks (AL) detected within the defined postoperative follow-up period, although we acknowledge the possibility of subclinical or late-presenting leaks may not have been captured, which has resulted in underestimation. Anastomotic leakage is multifactorial, and preservation of the inferior mesenteric artery (IMA) could not be reliably assessed in this cohort owing to the heterogeneous mix of benign and malignant cases. In benign disease, IMA preservation is more likely compared to oncological resections which often require high ligation for better lymph node count. Also, the impact of contemporary ‘three-row’ vs. ‘two-row’ circular stapler (CS) design on anastomotic leak rate is somewhat mixed as a large multicentre cohort analyses [30] showed ‘no-difference’ vs. a recent smaller study of similar comparison showed three-row circular stapler to be associated with lower AL rate and shorter LOS [31]. These device-specific factors should be interpreted alongside surgical technique, tissue perfusion, and patient-related risk factors.

Decisions regarding reversal, surgical approach, and timing were influenced by patient fitness, emergency status at index surgery, surgeon experience, and institutional practice rather than predefined criteria. Although multivariable analysis was performed, residual confounding from unmeasured variables - such as adhesional complexity, intraoperative decision-making, and surgeon skill & expertise - cannot be excluded. The obvious limitation of our study is the retrospective nature due to its inherent biases including information bias from incomplete records, institutional bias related to surgeon preference & local protocols and temporal bias due to evolving surgical techniques over the 13-year period. During this time, surgical practice, including laparoscopic expertise, perioperative care pathways such as ERAS, and patient selection may have evolved. Although we acknowledge this limitation, formal adjustment for year of surgery was not performed due to the relatively small number of events per year and the risk of model overfitting. As such, temporal trends could not be reliably quantified and residual confounding related to evolving surgical practice cannot be excluded. An important limitation of this study is the absence of patient-reported outcome measures, including quality of life, bowel function, and patient satisfaction following reversal. However, due to the retrospective design and the extended study period, such data were not routinely or consistently captured in a standardised format and were therefore not available for analysis. While we attempted to mitigate some of these biases through consistent data collection and statistical comparisons, the observational nature of the study inherently limits causal inference because of case selection and unmeasured confounding due to various patient and surgeon specific reasons.

Also, as a single-centre NHS study, these findings may not be generalisable to centres with different patient populations, resources, and operative practices. Despite these limitations, our study contributes real-world evidence from a small UK- based NHS hospital setting and highlights the importance of patient selection, fitness for surgery, and institutional practices in influencing Hartmann’s reversal rates and outcomes. These findings may help inform shared decision-making, resource allocation, and the design of future prospective or multi-centre studies.

## Conclusion

In conclusion, our single-centre retrospective study provides an overview of Hartmann’s procedure and reversal practices over a 13-year period in a small 450-bedded UK NHS hospital. We observed a relatively high reversal rate in younger, fitter patients with shorter LOS in laparoscopic group, with both groups showing acceptable short-term postoperative outcomes. However, these findings should be interpreted in context of the study’s limitations, including potential selection bias and confounding factors. Future prospective or randomised studies are needed to validate these findings and further explore the factors influencing Hartmann’s reversal.

## Data Availability

The datasets used and analysed for the current study are available from the corresponding author on reasonable request.

## Abbreviations

HP: Hartmann’s Procedure
HR: Hartmann’s Reversal
ASA: American Society of Anaesthesiologists
BMI: Body Mass Index
CCI: Charlson’s Co-morbidity Index
UK: United Kingdom
NHS: National Health Service
WHO: World Health Organization
LOS: Length of Stay
ERAS: Enhanced Recovery After Surgery
LRTI: Lower Respiratory Tract Infection
